# 1675. Community Acquired Serious Bacterial Infections in Pediatric Patients Before and After COVID: Could Masks Save Lives?

**DOI:** 10.1093/ofid/ofad500.1508

**Published:** 2023-11-27

**Authors:** Antonio C Arrieta, Stephanie Osborne, Delma Nieves, Matt Zahn, Adam Lee, Eric Shearer, Negar Ashouri

**Affiliations:** Children's Hospital of Orange County, Orange, California, USA, Orange, California; Children's Hospital of Orange County, Orange, California; CHOC Children's Hospital, Orange, California; Orange County Health Care Agency, Santa Ana, California; University of California Irvine, Orange, California; Orange County Health Care Agency (OCHCA), Orange, California; CHOC Children's Hospital, Orange, California

## Abstract

**Background:**

Association of serious bacterial infections (SBI) and preceding respiratory viral illness (RVI) has been described. Social distancing, (masking and lockdowns), in response to pandemic resulted in > 95% decrease in RVI, specifically influenza (Flu). Invasive *Streptococcus pneumoniae* (iSpneumo) and *Streptococcus pyogenes* (iGABHS) have substantial morbidity and mortality. Decrease in culture (Cx) proven iSpneumo during COVID has been reported. We used PCR in empyema fluid for enhanced SBI microbiologic diagnosis. Here, we evaluate Cx/PCR diagnosed community acquired SBI (CA-SBI) in children and relationship with Flu burden and social distancing.

**Methods:**

Data from surveillance of bacteremia and CA-empyema (Cx and PCR) at our institution was used to identify CA-SBI cases in children 3 months – < 18 years. Spinal fluid Cxs were reviewed to identify meningitis cases. We used data from the Orange County Health Care Agency for detection of countywide Flu trends. We normalized yearly CA-SBI cases per 10,000 discharges, grouped them into before COVID (BC) (7/1/2017 – 12/31/19) and after covid (AC) (1/1/2020 – 6/30/2022), and compared to countywide Flu cases 7/1/2017 – 6/30/2022.

**Results:**

We identified 361 CA-SBIs. *S aureus* (95; 26.3%) and *E coli* (65, 18%) were most common overall (Fig 1) and per cohort (BC 45; 25.7% and 25; 14.3% / AC 50; 26.9% and 40; 21.5%) respectively (Fig 2); 35.7% and 12.9% of iSpneumo and iGABHS respectively were identified by PCR only. Both decreased during COVID (iSpneumo BC 40; 22.9% to AC 16; 8.6% and iGABHS BC 24; 13.7% to AC 7; 3.8%). iSpneumo and iGABHS mortality was 3.6% and 12.9% respectively. There were no iSpneumo (5/2020 – 10/2021) or iGABHS 12/2020 – 11/2021) cases or deaths when Flu was not circulating.

**Figure:**
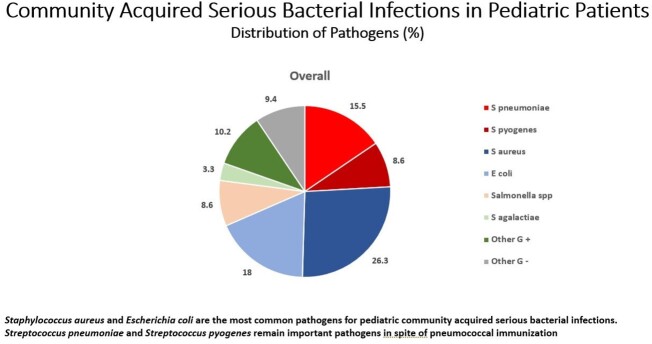

**Figure:**
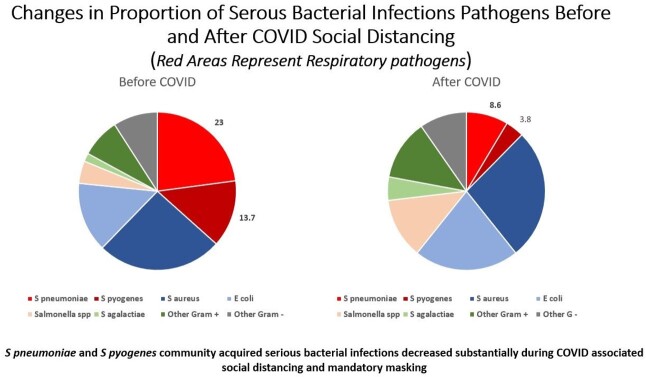

**Figure:**
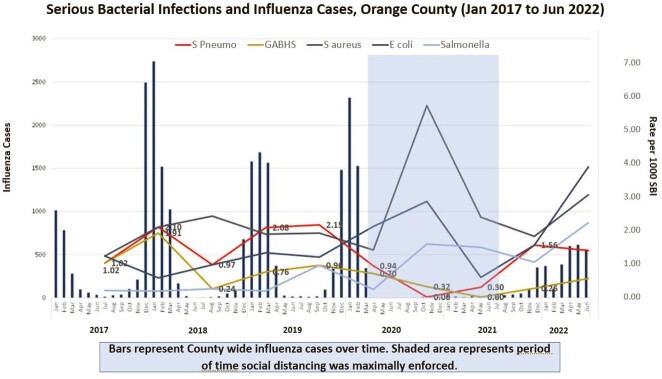

**Conclusion:**

iSpneumo and iGABHS cases decreased during social distancing, which included mandatory masking. No cases or deaths occurred when Flu was not circulating supporting association with Flu. Decrease in iSpneumo by routine pediatric vaccination has limitations due to differential protection by serotype and serotype replacement. Seasonal masking should be evaluated to decrease CA-SBI associated pediatric morbidity and mortality due to iSpneumo and iGABHS, further Flu vaccine development is needed.

**Disclosures:**

**Antonio C. Arrieta, MD, FIDSA, FPIDS**, Astellas Pharma Global Development, Inc.: Advisor/Consultant|Astellas Pharma Global Development, Inc.: Grant/Research Support|Astellas Pharma Global Development, Inc.: Honoraria|Cumberland Pharmaceutical: Grant/Research Support|IDbyDNA: Advisor/Consultant|IDbyDNA: Grant/Research Support|Melinta: Grant/Research Support|Merck: Advisor/Consultant|Merck: Grant/Research Support|Nabriva: Grant/Research Support|Paratek Pharmaceuticals: Grant/Research Support|Pfizer, Inc: Advisor/Consultant|Pfizer, Inc: Grant/Research Support|Roche/Genentech: Grant/Research Support|The Medicine Company: Grant/Research Support **Delma Nieves, MD**, Merck: Grant/Research Support|Summit Therapeutics: Grant/Research Support **Negar Ashouri, MD**, Merck: Grant/Research Support

